# Systems Biology Guided Gene Enrichment Approaches Improve Prediction of Chronic Post-surgical Pain After Spine Fusion

**DOI:** 10.3389/fgene.2021.594250

**Published:** 2021-03-23

**Authors:** Vidya Chidambaran, Valentina Pilipenko, Anil G. Jegga, Kristie Geisler, Lisa J. Martin

**Affiliations:** ^1^Department of Anesthesiology, Cincinnati Children’s Hospital Medical Center, Cincinnati, OH, United States; ^2^Division of Human Genetics, Cincinnati Children’s Hospital Medical Center, Cincinnati, OH, United States; ^3^Department of Pediatrics, University of Cincinnati College of Medicine, Cincinnati, OH, United States; ^4^Department of Biomedical Informatics, Cincinnati Children’s Hospital Medical Center, Cincinnati, OH, United States

**Keywords:** systems biology, genetics, polygenic risk score, chronic post-surgical pain, gene enrichment

## Abstract

**Objectives:**

Incorporation of genetic factors in psychosocial/perioperative models for predicting chronic postsurgical pain (CPSP) is key for personalization of analgesia. However, single variant associations with CPSP have small effect sizes, making polygenic risk assessment important. Unfortunately, pediatric CPSP studies are not sufficiently powered for unbiased genome wide association (GWAS). We previously leveraged systems biology to identify candidate genes associated with CPSP. The goal of this study was to use systems biology prioritized gene enrichment to generate polygenic risk scores (PRS) for improved prediction of CPSP in a prospectively enrolled clinical cohort.

**Methods:**

In a prospectively recruited cohort of 171 adolescents (14.5 ± 1.8 years, 75.4% female) undergoing spine fusion, we collected data about anesthesia/surgical factors, childhood anxiety sensitivity (CASI), acute pain/opioid use, pain outcomes 6–12 months post-surgery and blood (for DNA extraction/genotyping). We previously prioritized candidate genes using computational approaches based on similarity for functional annotations with a literature-derived “training set.” In this study, we tested ranked deciles of 1336 prioritized genes for increased representation of variants associated with CPSP, compared to 10,000 randomly selected control sets. Penalized regression (LASSO) was used to select final variants from enriched variant sets for calculation of PRS. PRS incorporated regression models were compared with previously published non-genetic models for predictive accuracy.

**Results:**

Incidence of CPSP in the prospective cohort was 40.4%. 33,104 case and 252,590 control variants were included for association analyses. The smallest gene set enriched for CPSP had 80/1010 variants associated with CPSP (*p* < 0.05), significantly higher than in 10,000 randomly selected control sets (*p* = 0.0004). LASSO selected 20 variants for calculating weighted PRS. Model adjusted for covariates including PRS had AUROC of 0.96 (95% CI: 0.92–0.99) for CPSP prediction, compared to 0.70 (95% CI: 0.59–0.82) for non-genetic model (*p* < 0.001). Odds ratios and positive regression coefficients for the final model were internally validated using bootstrapping: PRS [OR 1.98 (95% CI: 1.21–3.22); β 0.68 (95% CI: 0.19–0.74)] and CASI [OR 1.33 (95% CI: 1.03–1.72); β 0.29 (0.03–0.38)].

**Discussion:**

Systems biology guided PRS improved predictive accuracy of CPSP risk in a pediatric cohort. They have potential to serve as biomarkers to guide risk stratification and tailored prevention. Findings highlight systems biology approaches for deriving PRS for phenotypes in cohorts less amenable to large scale GWAS.

## Introduction

Chronic post-surgical pain (CPSP) is an underrecognized and undertreated problem with an incidence of 14.5–38% in children after major surgery, that significantly contributes to prolonged opioid use (Kain et al., 1996; Kehlet et al., 2006; Macrae, 2008; Rabbitts et al., 2017; Harbaugh et al., 2018). CPSP is defined as chronic pain that develops or increases intensity after a surgical procedure and persists beyond healing—at least 3 months after surgery (Werner and Kongsgaard, 2014). It has been recognized as a unique pain state recently in the International Classification of Diseases (ICD-11) (Schug et al., 2019). Chronic pain in adolescents leads to chronic pain in adults, imposes extraordinary annual costs on the health care system (Walker et al., 2010; Parsons et al., 2013), and negatively impacts physical and psychological health, leading to disability and depression (Hunfeld et al., 2001; Kashikar-Zuck et al., 2001; Fletcher et al., 2011). Hence, targeted, individualized preventive and therapeutic measures are needed to decrease CPSP occurrence. Development of such measures is impeded by the inability to accurately predict individual risk for CPSP.

Our previous studies investigating psychological and perioperative factors influencing pediatric CPSP showed that acute postoperative pain, surgical duration and psychological factors, such as those measured by the Childhood anxiety sensitivity index (CASI), are associated with CPSP risk in adolescents undergoing spine surgery (Chidambaran et al., 2017). However, these factors only explain 16% of variability in predicting CPSP, with medium accuracy (C-statistic 0.77). Thus, more accurate and objective biomarkers are needed to guide CPSP prevention and management.

Pain has a heritable component of up to 60%, suggesting incorporation of genetic factors may improve CPSP risk prediction. Our recent systematic literature review of genetic associations with CPSP (Chidambaran et al., 2019) showed that variants of several genes are associated with CPSP. However, any single variant had only a small effect size (Hoofwijk et al., 2016; Chidambaran et al., 2019). Since small effect sizes of single variants explain only a low percentage of the phenotypic variance, any one variant will not be useful at predicting risk. However, as individuals may harbor many variants each contributing modestly to risk, creating a risk score which accounts for the cumulative effect polygenic risk score (PRS) of many variants may better explain risk. PRS profiling has been shown to have translational potential as predictive, prognostic biomarkers (Muranen et al., 2016; Torkamani et al., 2018). Typically, the PRS builds off of the results of genome wide association studies (GWAS), whereby an individual’s genetic risk is the sum of all their risk alleles weighted by significance of the corresponding allele (Andersen et al., 2017; Escott-Price et al., 2017). Accurate, generalizable PRS have shown potential to inform clinical practice in several fields (Torkamani et al., 2018; Sugrue and Desikan, 2019). In fact, US Preventive Services Task Force recommended use of PRS for risk prediction and screening prioritization in prostate cancer (Grossman et al., 2018). There is also a push to incorporate PRS in risk assessment for decision-making in cardiovascular disease, breast cancer and Alzheimer’s disease (Maas et al., 2016; Knowles and Ashley, 2018; Tan et al., 2018). Richardson et al. (2019) used using UK Biobank data to analyze 162 GWAS-derived PRS for 551 heritable traits, and created an easily accessible web application—“An atlas of polygenic burden associations across the human phenome.” Pain was not identified as a phenotype in this atlas.

While CPSP is an important clinical problem the lack of GWAS studies related to pediatric CPSP to inform PRS is a major barrier. The problem is there are no pediatric biobanks to our knowledge with this phenotype. Additionally, pediatric clinical cohorts with well characterized CPSP phenotypes that are adequately powered to achieve GWAS statistical significance are difficult to recruit as they must have surgery and long-term follow-up. Given the lack of GWAS based data and the likelihood of small effect sizes, additional approaches to deriving PRS are required for pediatric CPSP. We recently described a systems-biology approach to identify genes and genetic pathways involved in CPSP (Chidambaran et al., 2020). This approach allows prioritization of functionally associated genes, hence substantially decreases the burden of statistical power for gene association testing and overcomes sample size limitations. We hypothesized that combining systems biology with gene enrichment for associated variants will allow derivation of PRS, which will improve prediction of CPSP risk in conjunction with known psychosocial factors. Our research is unique and novel, and lays the foundation for further research of PRS as predictive biomarkers of chronic pain conditions and less accessible cohorts (Tracey et al., 2019).

## Materials and Methods

This genomics study has two components: the first being a bioinformatics-driven, systems-biology approach to identify, rank and prioritize new “candidate genes” associated with CPSP, followed by a gene enrichment and association study in a prospectively recruited surgical cohort with penalized regression for PRS generation and evaluation.

### Systems Biology Gene Prioritization

We previously conducted a literature-based systematic review of human clinical studies of genetic associations with CPSP. We conducted a search using electronic databases (including PubMed and MEDLINE) of full-text articles of human clinical studies (limited to English language—clinical trials, multicenter studies, observational studies, and twin studies reported between 01/2002 and 12/2017) evaluating genetic associations with CPSP (Chidambaran et al., 2019). We used the following search terms: (“postoperative pain” OR “postsurgical pain” OR “postoperative pain” OR “postsurgical pain” OR “postoperative analgesia” OR “postoperative opioid” OR “CPSP” OR “chronic postsurgical pain”) AND (genetic association OR polymorphism OR variant OR “genotype” OR “Genome wide association” OR “SNP”). We included 21 full-text articles evaluating associations of 69 unique variants/haplotype with CPSP. Of these, variants of 31 genes involved in neurotransmission, pain signaling, immune responses and neuroactive ligand–receptor interaction, were found to be associated with CPSP (Supplementary Table 1). The results of the literature review including description of studies, genes, variants and outcomes are detailed elsewhere (Chidambaran et al., 2019). Using the literature derived genes (*N* = 31) as “training genes,” we previously identified novel candidate genes based on their similarity scores (“guilt by association”) to the curated training genes using ToppFun application of the Transcriptome Ontology Pathway PubMed based prioritization of genes (ToppGene) Suite, a one-stop portal of computational software tools for gene enrichment (Chen et al., 2009). Pathways based on training and top 10% candidate genes associated with CPSP are described in detail elsewhere (Chidambaran et al., 2020).

**FIGURE 1:**
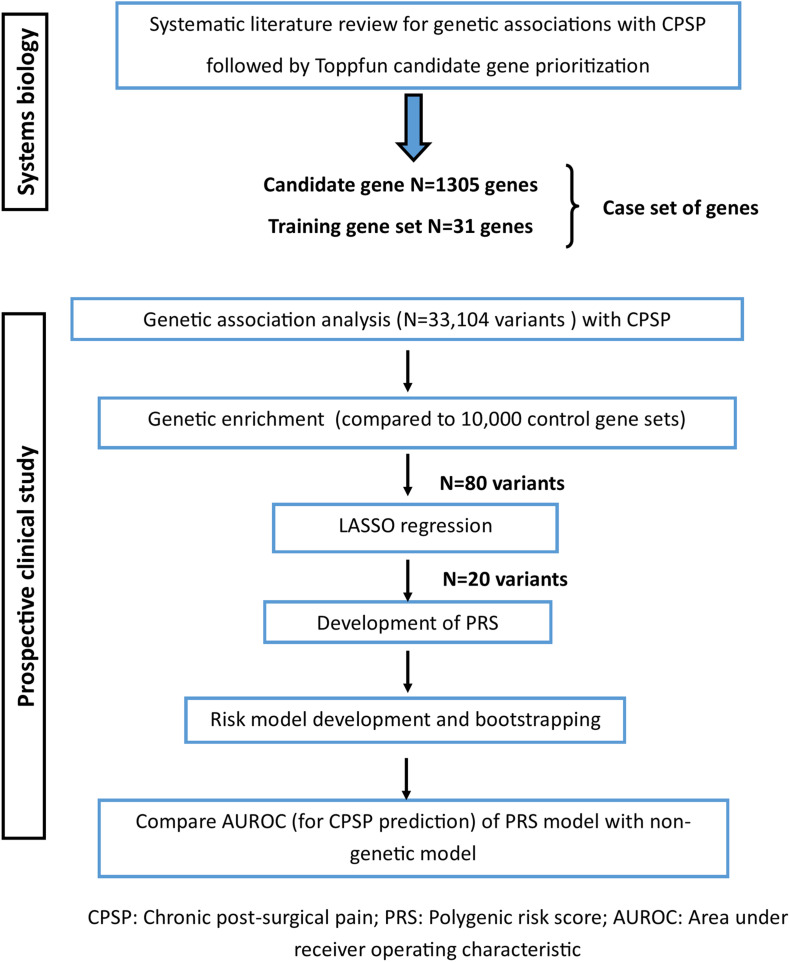
Study flow showing steps involved with gene prioritization using systems biology followed by genetic association analyses in the clinical cohort to derive polygenic risk score based prediction model for chronic post-surgical pain.

Here, as the next step, we used the curated training set (*N* = 31) and prioritized candidate genes (*N* = 1305) (henceforth referred to as the “case set” of genes) for association with and gene enrichment for CPSP in a prospective clinical cohort (Figure 1).

### Prospective Clinical Study

An observational prospective cohort study was conducted in adolescents with idiopathic scoliosis undergoing posterior spine fusion using standard surgical techniques, anesthetic and pain protocols. Studies are registered with ClinicalTrials.gov (Identifier: NCT01839461, NCT01731873), and approved by the Institutional Review Board. Written informed consent was obtained from parents and assent was obtained from children before enrollment.

#### Inclusion Criteria

Healthy children, age 10–18 years, American Society of Anesthesiologists (ASA) Physical Status ≤ 2 (mild systemic disease), diagnosis of idiopathic scoliosis and/or kyphosis, scheduled to undergo elective spinal fusion.

#### Exclusion Criteria

Pregnant or breastfeeding females, obesity, diagnosis of chronic pain or opioid use in the past 6 months, hepatic/renal disease and/or developmental delays.

#### Data Collection

Following preoperative data were obtained: demographics (sex, age, race), weight, pain scores (numerical rating scale/0–10 NRS) (von Baeyer, 2009) and home medications. Questionnaires were administered preoperatively to assess functional disability (FDI) (Walker and Greene, 1991) and anxiety sensitivity (CASI) (Silverman et al., 1991). All patients received total intravenous anesthesia (propofol and remifentanil) and midazolam in the intraoperative period, followed by standardized doses of patient controlled analgesia (morphine or hydromorphone) in the postoperative period. Pertinent surgical details (duration and number of vertebral levels fused) and anesthetic data (propofol and remifentanil doses) were collected. Postoperatively, pain scores (every 4 h), doses of morphine equivalents administered [postoperative days (POD) 1 and 2] were recorded. Of note, CASI, surgical duration and acute postoperative pain were associated with CPSP in a sub set of this cohort (Chidambaran et al., 2017). After hospital discharge, at 6–12 months, patients were asked to rate their average pain score (NRS) over the previous week and to answer open-ended questions about nature and site of pain, use of medications/alternative therapies/physician consults for pain, and functional disability (FDI).

#### CPSP Outcome

CPSP outcome was evaluated as a continuous variable for systems biology prioritization and predictive model development (to maximize power) and dichotomous outcome was used for comparison of predictive models. *CPSP continuous outcome*: Actual NRS pain scores at 6–12 months after surgery. *CPSP dichotomous outcome*, determined by pain score > 3/10 on a 11-point NRS (range 0–10) at 6–12 months after surgery (CPSP = yes) was used for final comparison of non-genetic versus PRS incorporated regression model to evaluate improvement in prediction characteristics. NRS for pain intensity has been validated as a pain measure in children aged 7–17 years (von Baeyer, 2009). NRS pain score > 3 (moderate/severe pain) at 3 months has been described as a predictor for persistence of pain and has been associated with functional disability (Gerbershagen et al., 2011).

#### DNA Collection and Genotyping

Blood was drawn for genotyping upon intravenous line placement. DNA was isolated on the same day, and frozen at −20°C. Genotyping was done using the Illumina Human Omni5 v41-0 array (85 patients), Human Omni5Exome v41-1 (33 patients) and Infinium Omni5-4-v1 (53 patients). Arrays were changed due to availability of new arrays which had more overall and more functional single nucleotide polymorphisms (SNPs).

#### Selection of Variants for Comparison of Case/Control Gene Sets

Only SNPs from autosomes were selected for analysis and were annotated using ANNOVAR software (Wang et al., 2010). All samples passed 95% threshold for call rates at genotype and individual levels. Genetic data was assessed for Hardy–Weinberg equilibrium (HWE) by means of goodness of fit χ^2^-test with threshold for *p*-values 0.0001 (Wang et al., 2010). SNPs that were not associated with a specific gene according to ANNOVAR annotation were excluded prior to analysis. Low-frequency variants (minor allele frequency less than 10%) were also excluded (Supplementary Figure 1). There were 4,186,587 variants on the exome chip initially and 542,313 variants remained after exclusion. SNPs in high linkage disequilibrium (LD) (80%) were pruned out in PLINK (Purcell et al., 2007) using the command –indep-pairwise 50 5 0.8.

#### Procedure for SNP Selection for PRS

The first step to identify SNPs associated with CPSP was genetic association analyses. The next step was to narrow down the number of significant SNPs by enrichment analysis. The last step for identifying SNPs included in PRS calculation was Least Absolute Shrinkage and Selection Operator (LASSO) regressions. SNPs with non-zero coefficients were selected for PRS.

##### Genetic Association Analyses

Analyses were conducted using SAS 9.4 (SAS, Cary, NC) and R^1^. Prior to genetic analyses, cryptic relatedness was checked using Graphical Representation of Relationship (GRR) (Abecasis et al., 2001). Principal component analysis was employed to confirm European and African continental ancestry using 482 validated ancestry informative markers (Tandon et al., 2011). Concordance with self-reported race was > 95%. Given the concordance, race was used as a covariate in all the models and not principal components. To identify significant SNPs, we used linear models for association of each SNP with CPSP continuous outcome. In all association tests, we used an additive genetic model in which major homozygotes were coded as 0, heterozygotes as 1, and minor homozygotes as 2. Univariate analyses were conducted for CPSP outcomes with initial covariates (demographics, surgical duration, CASI, anesthetic doses, preoperative pain score), as suggested by non-genetic covariates based on our previous findings in a similar cohort (Chidambaran et al., 2017). Covariates significant in univariate analyses (*p* < 0.1) were included for genetic association analyses. PLINK v.07 was used for genetic association tests. Since the association results are only relevant for comparing the significant variants within the ranked case gene sets and those within the control sets for enrichment, they are not reported separately.

##### Gene Enrichment Analyses

Case gene variants were analyzed as sequence of cumulative sums of ranked variant sets with 10% increment, as has been done in a prior study (Kurowski et al., 2019). The first addend in each sequence was the training gene variant set. For each cumulative sum, we compared the number of associations in our case sets that met the *p* < 0.05 threshold to the number of associations meeting the same criteria in 10,000 matched runs of our control set of genes. SNPs from the control set were selected in the same ratio for minor allele frequency (MAF) as it was observed in the case set. Specifically, we used MAF bands as follows: 10–15%:15–20%:20–30%:30–50%. Empirical *p*-values of resampling tests were computed as follows: we calculated how many samples out of 10,000 had the number of significant SNPs equal to or greater than the number of significant SNPs from the case set and divided this number by 10,000. SNPs in case genes that formed the earliest cumulative group (where the number of significant SNPs were greater than in the matched control group) were considered as a minimal set of variants enriched for associations with corresponding outcomes.

##### LASSO Regression

To minimize risk of overfitting, we used penalized regression with LASSO in R software (package glmnet) *after* enrichment analyses (Friedman et al., 2010) with CPSP continuous and categorical outcome. SNPs in the genes identified in enrichment analysis were considered for penalized regression. Since penalized regression can be performed only on data without missing values we imputed missing genotypes using Michigan Imputation Server^2^. We imputed chromosomes where SNPs with missing genotypes were located. For each chromosome we submitted two VCF (Variant Call Format) files for subset of white patients and for subset of blacks and with admixture patients. VCF files were obtained from PLINK files using PLINK v1.9. Submitted to the server SNPs had 100% call rate. Both QC and imputation modes were used at the server. Genotypes for subset of white patients were imputed against the 1000G Phase 3 reference panel and the second subset of patients was imputed against the CAAPA African American reference panel. SNPs of interest were extracted from the files with imputed genotypes received from the server. Since SNPs with imputed genotypes overlapped with non-missing genotypes of original data these two types of genotypes (original and imputed) were used for evaluation of imputation accuracy. A controlling penalty parameter lambda for penalized regression was selected via cross-validation approach.

##### PRS Calculation

SNPs with non-zero coefficients in LASSO model were selected for PRS calculation. PRS was calculated as a weighted sum of products between number of risk alleles and their corresponding regression coefficients. The mathematical formula used for PRS calculation was given by the following equation

$$P\,R\,S_{n}=\sum_{i=1}^{m}\left(\left|b_{i}\right|*R_{i,n}\right)$$

Where *i* is a number of SNPs, *m* is an upper range of SNPs participating in PRS calculation, *n* is a number of patients, *PRS*_*n*_ is a polygenic risk score for *n*-th patient, *b*_*i*_ is an absolute value of regression coefficient for each out of *m* SNPs from linear regression models for association of CPSP with a given SNP, R_*i,n*_ is number of risk alleles for *i*-th SNP for *n*-th patient.

#### Regression Models

We built logistic regression models using stepwise approach including significant non-genetic predictors associated with CPSP (*p* < 0.05 selection criteria), followed by inclusion of PRS. For model performances, we used the area under the receiver operating characteristics (ROC) curve (AUC). AUCs with 95% confidence intervals for clinical and genetic models were used for model comparison in SAS 9.4 (SAS., Cary, NC).

#### Bootstrapping

While the optimal design for validation is to use an independent sample for validation, given the challenges in collecting such samples, we used the bootstrap method to internally validate the prediction. In this method, new bootstrap samples are generated from the original sample by random drawing with replacement multiple times (Efron, 1979). By bootstrapping across many iterations, the accuracy of parameter estimates can be determined. In this study, we empirically evaluate bias in the regression coefficients from the original model. Bootstrapping bias is a difference between the value obtained by using the original sample and the mean value obtained using bootstrap samples. At each iteration (*n* = 1,000), a random bootstrap sample (the same size as the original sample) was drawn with replacement from the original sample. Logistic models were generated for each bootstrap sample and bootstrapping results were compared with results from the original model. Regression coefficients and bootstrap confidence intervals are reported as linear terms and equivalent odds ratios. Bootstrapping was performed in R software (R Core Team, 2018) with the package boot (Davison and Hinkley, 1997; Freeman, 1998).

#### Power Analysis

For the gene set enrichment analyses, our goal was to determine if a set of selected genes/variants were more likely to show association (*p* ≤ 0.05) than for a set of variants selected by chance. Out of 33,104 variants, we created deciles of variants, and the rates of associated variants compared each decile to 10,000 randomly selected gene sets of equal size. Based on the one sided proportion test, if we assumed that the background rate for association in the random set was 0.05, in the first decile containing 3310 SNPs, we would have 80% power to detect a difference between the SNPs in the selected genes if they were associated at a rate of 0.064 (OR = 1.3) at alpha = 0.05. Notably, the power calculation for gene enrichment was based on the number of SNPs rather than the number of individuals in the sample because we are comparing the rates of SNPs nominally associated between selected genes and random genes. For individual variants, we would have 80% power to detect an odds ratio as small as 2.1 at alpha = 0.05 and minor allele frequency 0.4. To evaluate the PRS risk score, we evaluated the score in 52 individuals with CPSP and 79 individuals without CPSP. With these numbers we would have 80% power to detect a PRS score difference as small as 2 at alpha = 0.05.

## Results

### Prospective Cohort Characteristics

Demographics and summary of the variables examined for the prospective cohort are listed in Table 1. CPSP outcome was determined for 131 of the 171 patients (∼23% loss to follow-up). The flow diagram for recruitment is presented in Supplementary Figure 2. We examined the characteristics of both cohort of subjects lost to follow-up and the cohort of subjects followed for 6–12 months for all pertinent measures included in the models and did not find significant differences in terms of age (*p* = 0.390), sex (*p* = 0.361), race (0.906), CASI (*p* = 0.364), surgical duration (*p* = 0.322) and preoperative pain (*p* = 0.879). We found a 40.4% (53/131) incidence of CPSP. CPSP cases had significantly higher preoperative pain scores (*p* = 0.037) and CASI (*p* = 0.003) on univariate analyses and these factors were included as predictors in the regression model, and covariates for genetic association analyses.

**TABLE 1:** Baseline and pain follow-up characteristics of the surgical cohort, based on chronic post-surgical outcomes and univariate analyses of perioperative/psychological covariates.

**Variable**	**Entire cohort (*N* = 171)**		**CPSP (dichotomous outcome)**		***p*-value**	**Pain score at 6–12 months (continuous outcome)**	
			**CPSP Yes (*N* = 53)**	**CPSP No (*N* = 78)**			
	
**Demographics**						**Median (IQR)**	*p*-value*
Sex F%	75.4%		81.0%	74.4%	0.365	2 (0–4)	0.331
Sex M%	24.6%		19.0%	25.6%		0 (0–4)	
Race (White %)	81.8%		77.4%	84.6%	0.292	1 (0–4)	0.844
Race (Non-white %)	18.2%		22.6%	15.4%		3 (0–4)	

	**Mean**	***SD***	**Mean (*SD*)**	**Mean (*SD*)**	***p*-value**	**Coefficient (*SE*)**	*p*-value**

Weight (Kg)	57.446	15.256	56.3 (14.2)	57.0 (14.5)	0.781	**−**0.055 (0.018)	0.323
Age (years)	14.488	1.840	14.7 (1.8)	14.5 (1.8)	0.462	0.184 (0.139)	0.189
**Preoperative characteristics**							
Preoperative pain score	0.596	1.282	0.3 (0.5)	0.1 (0.3)	0.037	1.210 (0.648)	0.065
CASI	28.552	5.531	30.6 (5.6)	26.8 (4.9)	0.003	0.147 (0.048)	0.003
**Surgical/anesthesia characteristics**							
Surgical duration	4.816	1.232	5.0 (1.4)	4.8 (1.2)	0.376	0.360 (0.214)	0.095
No. vertebral levels fused	11.506	1.969	11.0 (2.3)	11.6 (1.9)	0.115	0.006 (0.130)	0.963
Propofol dose mg/kg	71.791	27.186	79.5 (27.0)	73.7 (28.7)	0.238	0.014 (0.008)	0.091
Remifentanil dose mcg/kg	113.911	40.891	118.6 (41.5)	115.2 (44.2)	0.563	0.008 (0.006)	0.225
**Acute postoperative pain characteristics**							
AUC POD1–2	200.327	73.490	222.7 (75.9)	196.7 (66.8)	0.053	0.004 (0.003)	0.697
Morphine meq POD1–2 mg/kg	1.626	0.747	1.6 (0.7)	0.8 (0.1)	0.065	0.646 (0.349)	0.067
**Pain follow-up at 6–12 months**							
CPSP Y/No %	53/78 (40.5%)						
FDI score	4.485	5.321	6.7 (5.9)	2.3 (4.0)	0.002		
Pain score (NRS)	2.240	2.457	4.6 (2.0)	0.6 (1.0)	<0.001		

* Wilcoxon test. ** Simple linear regression. CASI, Childhood anxiety sensitivity index; AUC, Area under curve of pain scores over postoperative days (POD) 1 and 2; CPSP, Chronic post-surgical pain; FDI, Functional disability index.

### Genetic Enrichment

**FIGURE 2:**
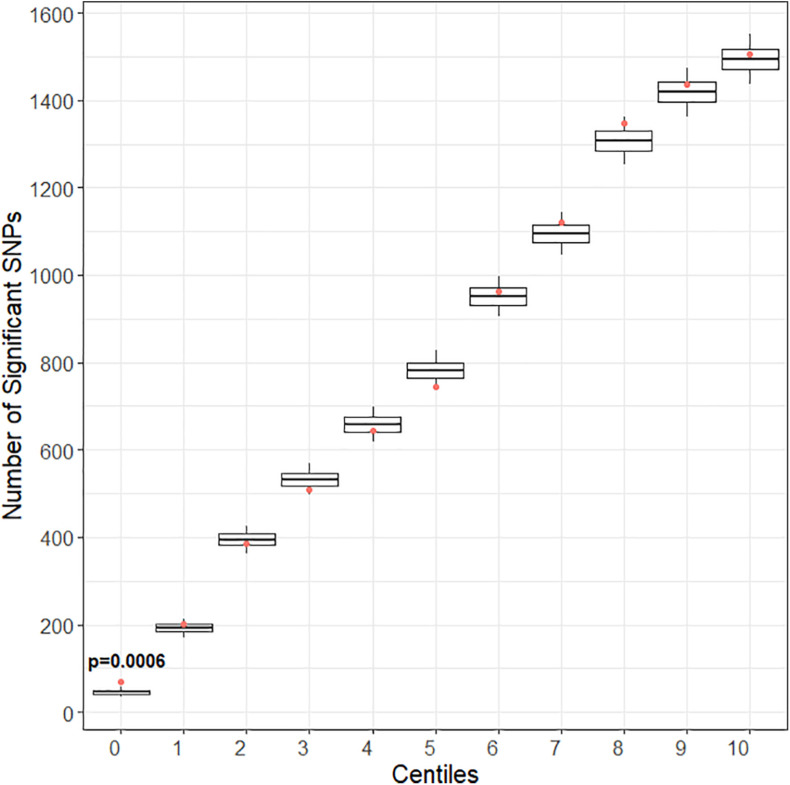
Gene enrichment analyses for pain score at 6–12 months as outcome. Centiles represent the portion of case genes used in the genetic assocaition analysis. 0% includes the training set of gene variants, 10th percentile includes the training list plus the top 10% highest ranked genes, and so forth, vertical axis represents the number of variants. Box plots represent the cumulative number of SNPs with signficant association with pain score at 6–12 months after surgery [chronic post-surgical pain (CPSP) continuous outcome] (*p* < 0.05) in 10,000 runs of control gene variants. The dot indicates the cumulative number of nominal associations (*p* < 0.05) identified for case genes. Enrichment is indicated when a greater number of genetic associations are present in case versus control genes, that is, when the number of associations in case genes (red dot) (80 variants/1010 variants) exceeded the upper 95th percentile threshold in the 10,000 runs of the control set. For CPSP continuous outcome, we see enrichment in the training set of variants (*p* < 0.001). The training set incudes 80 variants showing association with CPSP (*p* < 0.05).

After quality control and pruning as described under methods, 33,104 case variants and 252,590 control variants were included for covariate adjusted association analyses. Compared to the control set, there was enrichment of SNP associations in the training set for CPSP (Figure 2) but not for the other deciles of candidate gene variant sets. Of 1010 variants included in the training set, the number of variants (*N* = 80) associated with CPSP (*p* < 0.05) was significantly higher than in 10,000 randomly selected control sets (*p* = 0.0004). These 80 variants were annotated to the following 12 genes:ATXN1 (29); CACNG2 (2); CTSG (2); DRD2 (1); HLA-DQB1 (3); IL10 (1); KCNA1 (1); KCND2 (5); KCNJ3 (3); KCNJ6 (9); KCNK3 (2); PRKCA (22).

### LASSO

Before LASSO, we imputed 45 genotypes in all (16 individual SNPs over 26 patients, where missing genotypes ranged from 1 to 10 at an individual level). One SNP rs17843723 form the HLA-DQB1 gene failed imputation and was excluded from consequent analysis. Imputation accuracy was 100% when we compared genotypes detected by chips with imputed genotypes. Number of genotypes for imputation accuracy evaluation was 2,051 (131 patients ^∗^ 16 SNPs – 45 genotypes with missing values = 2,051 genotypes for accuracy evaluation). After LASSO, when CPSP was a continuous variable, the prediction set was comprised of 53 variants. LASSO regression with CPSP as a categorical variable resulted in 24 variants. We identified 20 variants that had non-zero coefficients in both linear and logistic penalized regression models. Chromosomal location, genetic annotation, function, MAF, odds ratios for CPSP and beta for NRS at 6–12 months with *p-*values for the LASSO selected variants are provided in Table 2. These 20 variants were annotated to nine genes: ATXN1 (7); CACNG2 (1); DRD2 (1); KCNJ3 (2); KCNJ6 (1); KCNK3 (1); PRKCA (7). Of these variants, rs7220480 was imputed for one individual, and rs2891519 and rs200369418 were imputed for three individuals.

**TABLE 2:** Genetic variants and risk alleles with regression coefficients included in the determination of polygenic risk score for prediction of chronic post-surgical pain.

**SNP**	**Observed major allele**	**Observed minor allele**	**Gene**	**#Linear regression weight**	***p*-value linear regression**	**Reference allele**	**Alternative allele**	**Function**	**Chr**	**Location (GRCh37)**	**Minor allele frequency**
rs62069959	G*	A	PRKCA	2.299	0.001	C	T	Intronic	17	64318923	0.196
rs7125415	G	A*	DRD2	1.657	0.034	C	T	Intronic	11	113000000	0.126
rs61131185	A	G*	ATXN1	1.524	0.011	A	G	Intronic	6	16623387	0.322
rs12665284	G*	A	ATXN1	1.481	0.041	G	A	Intronic	6	16626066	0.146
rs202146909	A*	G	KCNJ3	1.414	0.042	T	C	Intronic	2	156000000	0.193
rs493352	G*	A	ATXN1	1.242	0.031	T	C	Intronic	6	16744169	0.488
rs9754467	A*	G	CACNG2	1.166	0.032	G	A	Intronic	22	37019059	0.222
rs12198202	A*	G	ATXN1	1.064	0.005	T	C	Intronic	6	16679771	0.424
rs11079653	T*	A	PRKCA	0.98	0.011	A	T	Intronic	17	64352329	0.202
rs2850125	G*	A	KCNJ6	0.936	0.046	C	T	Intronic	21	39130114	0.456
rs9914723	G	A*	PRKCA	0.917	0.004	G	A	Intronic	17	64716397	0.196
rs7220480 ^1^	A	G*	PRKCA	0.857	0.048	A	G	Intronic	17	64686679	0.406
rs2891519 ^2^	G	A*	KCNK3	0.835	0.008	G	A	Downstream	2	26954991	0.220
rs200369418 ^2^	A*	C	PRKCA	0.816	0.028	C	A	Intronic	17	64762496	0.500
rs3812204	G	A*	ATXN1	0.789	0.038	G	A	Intronic	6	16698022	0.345
rs4716060	C	A*	ATXN1	0.772	0.038	C	A	Intronic	6	16310456	0.345
rs6459476	A	C*	ATXN1	0.736	0.048	A	C	Intronic	6	16618187	0.348
rs227912	A*	G	PRKCA	0.678	0.049	G	A	Intronic	17	64610729	0.246
rs744214	G*	A	PRKCA	0.634	0.017	G	A	Intronic	17	64334856	0.316
rs1992701	G	A*	KCNJ3	0.584	0.047	C	T	Intronic	2	156000000	0.453

PRKCA (protein kinase C alpha); DRD2 (dopamine receptor D2); ATXN1 (ataxin 1); KCNJ3 (potassium voltage-gated channel subfamily J member 3); CACNG2 (calcium voltage-gated channel auxiliary subunit gamma 2); KCNJ6 (potassium voltage-gated channel subfamily J member 6); KCNK3 (potassium two pore domain channel subfamily K member 3). #Linear regression coefficients were used to calculate weighted polygenic risk scores; Beta > 0 is the selection criteria per LASSO. *Risk allele; 1—imputed for one patient; 2—imputed for 3 patients.

### Polygenic Risk Scores

**FIGURE 3:**
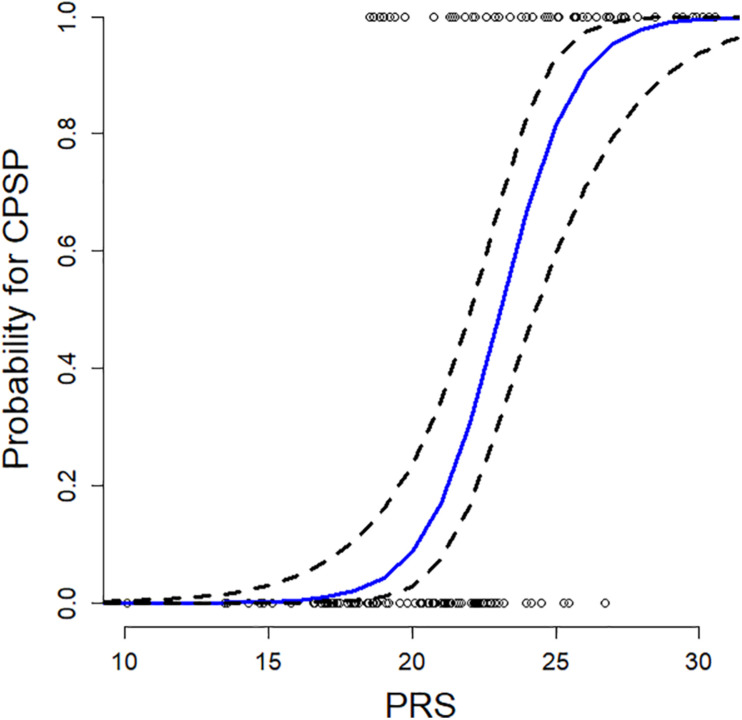
Plot of predicted probability of developing chronic postsurgical pain (CPSP) after spine surgery is presented as a function of polygenic risk score (PRS), at a childhood anxiety sensitivity index (CASI) score of 28.3 (median CASI in the model). The blue line denotes predicted probabilities from the final regression model, and dashed lines the 95% confidence interval, and circles represent observed cases (or outcomes). We see a sigmoid shaped curve with increasing probability of CPSP at PRS > 16, 50% probability at PRS = 23.06 and high probability beyond PRS = 30. Thus, higher the weighted PRS, higher the probability of CPSP.

Weighted genetic risk was calculated from the 20 SNPs selected by LASSO regression models. PRS ranged from 10.1 to 30.6 (mean: 21.1; SD 4.0) and were normally distributed. The predicted probability (with 95% CI) of CPSP for a subject having a median (for the cohort) CASI = 28.3 using the regression model is plotted as a function of the PRS in Figure 3. The probability of CPSP is higher than 50% at a PRS > 23.06.

### Regression Models

**FIGURE 4:**
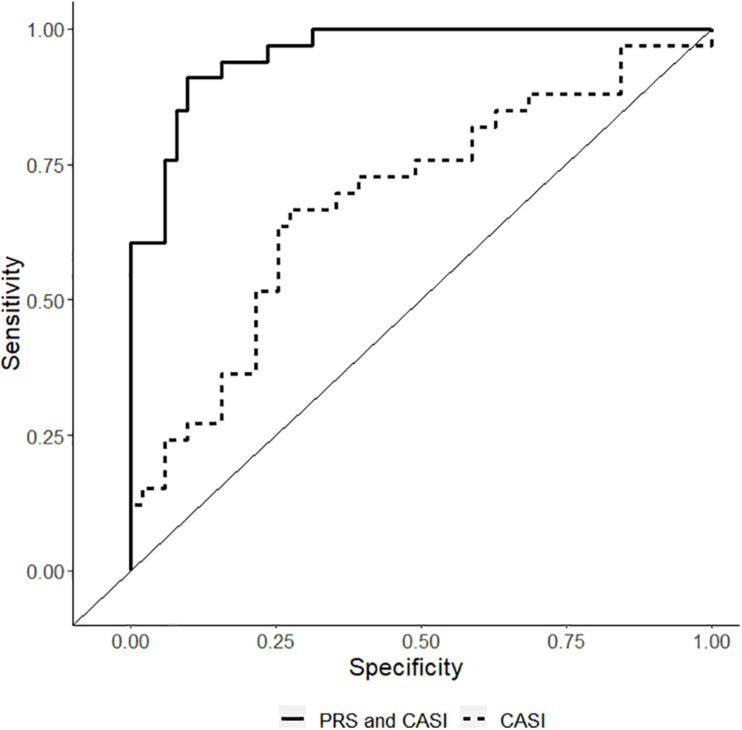
Receiver operating characteristic curve showing the sensitivity/1-specificity for prediction of chronic post-surgical pain using the non-genetic model [including childhood anxiety sensitivity index (CASI) – dashed lines] compared with the prediction using the polygenic risk score final model (PRS and CASI – solid black lines). The area under curve for genetic model is 0.96 (95% CI: 0.92–0.99) compared to 0.70 (95% CI: 0.59–0.82) for non-genetic model (*p* = 0.0001).

The non-genetic full and reduced model are presented in Table 3. The genetic model incorporating PRS in the non-genetic reduced model is also presented in Table 3. In the final model, both CASI and PRS remained significant predictors with Odds ratio (OR) of 1.37 (95% CI: 1.15–1.65) and 2.16 (95% CI: 1.53–3.05), respectively, for CPSP. In the final model, regression coefficients for CASI and PRS have means and standard errors for linear terms 0.32 ± 0.09 and 0.77 ± 0.18, respectively. Comparison of performance of the predictive model with clinical predictor (CASI) and performance of the predictive model with PRS (PRS and CASI) showed statistically significant higher performance of genetic model. Receiver operating characteristic curve was plotted showing that AUC for genetic model was 0.96 (95% CI: 0.92–0.99) compared to 0.70 (95% CI: 0.59–0.82) for non-genetic model (*p* = 0.0001) (Figure 4).

**TABLE 3:** Multiple regression models evaluated for prediction of chronic post-surgical pain (CPSP) and results of bootstrapping.

**Independent variable**	**OR**	**Lower 95% CI**	**Upper 95% CI**	***P*-values**
**Full clinical model (AUC = 0.71)**				
CASI	1.15	1.04	1.25	0.0038
Preoperative Pain	1.40	0.45	4.33	0.5559
**Reduced clinical model (AUC = 0.70)**				
CASI	1.15	1.04	1.26	0.0035

**Genetic model (AUC = 0.96)**				

**Independent variable**	**OR**	**Lower 95% CI**	**Upper 95% CI**	***P*-values**

CASI	1.37	1.15	1.65	0.0006
Weighted PRS	2.16	1.53	3.05	<0.0001

**Bootstrapping results**				

	**OR (β)**	**Lower 95%CI, OR (β)**	**Upper 95%CI OR (β)**	**Bias β**

CASI	1.33 (0.29)	1.03 (0.03)	1.72 (0.38)	0.03
Weighted PRS	1.98 (0.68)	1.21 (0.19)	3.22 (0.74)	0.09

*CASI, Childhood anxiety sensitivity index; OR, Odds ratio; β, regression coefficients; AUC, Area under curve; PRS, Polygenic risk score; CI, confidence interval.*

### Bootstrapping

The final predictive model was evaluated by bootstrapping. Bootstrapping bias for means of linear terms were positive values for both CASI (0.03) and PRS (0.09) with standard errors 0.13 and 0.25 for means 0.29 and 0.68, respectively. Thus, bootstrap means for linear terms for CASI were 0.29 (0.32 minus 0.03) with 95% confidence interval 0.03–0.38 and for PRS 0.68 (0.77 minus 0.09) with 95% confidence interval 0.19–0.74. Confidence intervals for each regression coefficient obtained using bootstrapping serve as assessments for the model prediction accuracy. OR and 95% CI for CASI and PRS after bootstrapping remained similar to initial model results at 1.33 (95% CI: 1.03–1.72) and 1.98 (1.21–3.22), respectively. Bootstrapping bias means of linear terms, corresponding ORs with 95% CIs for regression coefficients are given in Table 3.

## Discussion

For phenotypes affected by difficulties in recruiting well powered and well characterized cohorts, novel methodologies are needed to address gaps in objective and accurate predictors. This is especially true for pediatric CPSP as it impedes targeted preventive efforts. By leveraging systems-biology and genetic testing approaches, we conducted enrichment analyses to derive PRS. They were calculated as weighted sum of products between number of risk alleles at 20 variants selected by LASSO regression, and their corresponding regression coefficients. We used bootstrapping to validate our final model’s performance. Two factors—PRS and CASI—remained in the final risk model which predicted CPSP with higher accuracy compared to base non-genetic model (*p* = 0.0001). Since CPSP is a biopsychosocial phenomenon, it is not surprising that CASI, a psychological construct that measures interpretation of anxiety-related symptoms, remained a major risk predictor along with PRS. Higher anxiety sensitivity is associated with fear of pain, pain interference, which then leads to increased avoidance, disability (Martin et al., 2007) and maladaptive coping styles (Asmundson and Taylor, 1996), thus leading to the persistence of pain. Preoperative assessment of CASI will allow interventions such as education for improved coping, behavioral therapy and possibly use of anti-anxiolytics to temper the pain experience.

Scarcity of available genomic datasets for our phenotype of interest, namely, CPSP, makes GWAS daunting. Systems-biology approaches have been used successfully for identifying gene pathways implicated in other phenotypes (Kurowski et al., 2012; Jegga, 2014; Kurowski et al., 2019) as they allow leveraging known genomic data sources to prioritize functional genes for association, thereby decreasing the statistical burden. In our study, literature derived training sets showed enrichment for CPSP, with genes previously known to play an important role in pain. This either suggests that all relevant genes have been captured by the studies so far or that there are additional genes in very different pathways which need additional larger studies. Importantly, systems biology helped us identify control gene sets which allowed us to refine the optimal variants for PRS determination by enrichment. Our findings are an important first step in the development of accurate and reliable gene-based biomarkers to predict susceptibility for CPSP. However, these findings will need external validation in unrelated similar and dissimilar surgical cohorts and diverse population structures. In addition, analytic validation of the panel in a CLIA-certified laboratory by re-sequencing and confirmation of the variants is necessary. Nevertheless, there is promising potential for future automated risk decision support based on preemptive genotyping and patient characteristics (CASI). This will allow preemptive preventive strategies to be employed cost-effectively, directed at those with higher risk.

The derived PRS is composed of weighted risk coefficients from 20 variants annotated to 7 genes which (not surprisingly) played a role in CPSP in previous studies: Ataxin-1 (*ATXN1)*, Protein Kinase C Alpha (*PRKCA)*, calcium channel genes (codes for the G subunit: *CACNG2)*, Dopamine receptor gene (*DRD2*) and potassium channel genes (*KCNJ3, KCNJ6, KCNK3)*. Potassium and calcium channel genes form the majority of genes involved. This is consistent with knowledge that these channels contribute to activation thresholds and spontaneous or exaggerated neuronal firing in response to noxious stimuli (Cohen and Mao, 2014). CPSP risk 6 months after breast cancer surgery has previously been reported for haplotype A2 rs3111020-rs11895478 G-A of *KCNJ3* and rs2835925 of *KCNJ6* (Langford et al., 2015). Similarly, in another cohort, several variants of the *CACNG2* gene were found to be associated with CPSP at a nominal level after breast cancer surgery (Nissenbaum et al., 2010). *PRKCA* is involved in long-term potentiation, an important process for memory and chronic pain development (Kawasaki et al., 2004; Price and Inyang, 2015). A meta-analysis showed that a recessive model of allele A in rs887797 in *PRKCA* was strongly associated with neuropathic CPSP in adults undergoing joint replacement surgery (Warner et al., 2017). *DRD2* variants were nominally associated with CPSP 4 months after different surgeries (Montes et al., 2015), as well as in chronic pain conditions (migraine) and substance abuse/addiction (Xu et al., 2004; Connor et al., 2007; Todt et al., 2009). Ataxin1 (*ATXN1*) is a gene that may play a role in transcription. Although its role in pain is not known, a study of a multiple surgery cohort found that the A allele at rs179997 of *ATXN1*was associated with CPSP at 4 months (Montes et al., 2015). Although variants selected for PRS in our study are mostly intronic, a functional assessment of the variants informing the PRS is not pertinent for establishing predictive biomarkers. However, intronic sequence alterations could influence gene function via altering binding sites for splicing co-factors or transcriptional enhancer/suppressor elements or may be in linkage with other variants with functional roles.

Since different surgeries are associated with variable pain modalities with different incidences of CPSP, the homogeneity of the surgical cohort in our study is a strength. The well characterized CPSP phenotypes, systematic approaches and bootstrapping add to the robustness of the results. Recent articles discuss clinical implementation of PRS may soon be a reality in cohorts with a higher prior probability of disease, to assist in risk/diagnosis or to inform treatment choices (Lewis and Vassos, 2020). We acknowledge that there are ethical and scientific challenges surrounding clinical implementation of PRS (Martin et al., 2019). Cost-benefit analyses for use of PRS in CPSP will need to consider (a) the prevalence of cohort at risk (In the US alone, 25 million adult and 5 million pediatric major surgeries are conducted per year (specifically, for spine surgery— according to the national scoliosis foundation, about 38,000 spine fusions are conducted in idiopathic scoliosis every year in the United States) (Sieberg et al., 2013) (b) the relative risk of phenotype predicted by PRS (in this study, RR∼2.2), (c) the proportion of surgical population at risk (in this study, ∼40%; the incidence of severe CPSP after major surgery is 2.2%—at a conservative estimate, this translates to 660,000 new cases of CPSP every year in the United States) (Fletcher et al., 2011), (d) the therapeutic response rate (CPSP is potentially preventable), and (e) the cost/impact of the condition being prevented (Gibson, 2019). Recent estimates suggest that CPSP incurs annual direct and indirect costs of US$11,846 and US$29,617, respectively, per patient (Parsons et al., 2013) and negatively impacts quality of life (Hunfeld et al., 2001; Kashikar-Zuck et al., 2001; Fletcher et al., 2011). Furthermore, the decreasing costs of genetic testing indicate that use of PRS will have benefits that outweigh risks/costs. Recent studies investigating preventive strategies like pregabalin have conflicting results (Mishriky et al., 2015; Thapa and Euasobhon, 2018)—this is not necessarily a function of therapeutic inefficacy—but could potentially be due to bias from inclusion of low risk subjects; hence, PRS could potentially improve evaluation of interventional strategies allowing *a priori* assessment of risk.

## Conclusion

In conclusion, systems biology approaches combined with genetic association testing methodology are useful methods to develop PRS when GWAS approaches are not feasible. PRS holds future potential as a biomarker (simple blood test) that can predict CPSP risk. Given the morbidity associated with CPSP—including the risk for opioid abuse (Brummett et al., 2017), significant rates of chronic opioid dependence after surgery (Lee et al., 2017), the economic burden of CPSP—and decreasing genetic testing costs, we envision PRS to be cost-effective adjunct for risk stratification and clinical decision-making so preventive strategies can be targeted at those with high-risk. Future studies are needed to validate our findings. Our results may also have extended potential in predicting other chronic musculoskeletal pain conditions with similar pathophysiology.

## Data Availability Statement

The datasets generated for this study can be found in the dbGaP production site at this url: http://www.ncbi.nlm.nih.gov/projects/gap/cgi-bin/study.cgi?study_id=phs002105.v1.p1.

## Ethics Statement

The studies involving human participants were reviewed and approved by Cincinnati Childrens Institutional Review Board. Written informed consent to participate in this study was provided by the participants’ legal guardian/next of kin.

## Author Contributions

VC developed the research concept, oversaw data collection, recruitment, data analyses, and wrote the first draft of the manuscript. VP and LM conducted the statistical genetics analyses and revised the manuscript. AJ conducted the systems biology modeling. KG was the research coordinator who approached and recruited subjects for the prospective data analyses. All authors contributed to the article and approved the submitted version.

## Conflict of Interest

The authors declare that the research was conducted in the absence of any commercial or financial relationships that could be construed as a potential conflict of interest.

## Footnotes

1 http://www.R-project.org

2 https://imputationserver.sph.umich.edu
