# Supplementary material for: Systems Biology Guided Gene Enrichment Approaches Improve Prediction of Chronic Post-surgical Pain After Spine Fusion
Source: Front Genet. 2021 Mar 23;12:594250. doi: 10.3389/fgene.2021.594250 (PMC8044807; doi:10.3389/fgene.2021.594250)
Supplement: Supplementary file 1 [file Data_Sheet_1.docx]

**Supplementary section**

**Systems biology guided gene enrichment approaches improve Prediction of Chronic Post-Surgical Pain after Spine Fusion**

Vidya Chidambaran^1,2^, Valentina Pilipenko^3^, Anil G. Jegga, PhD^2,4^, Kristie Geisler^1^, Lisa J. Martin^2,3^

^1^Department of Anesthesiology, Cincinnati Children’s Hospital Medical Center, Cincinnati, OH, USA

^2^Department of Pediatrics, University of Cincinnati College of Medicine, Cincinnati, OH, USA

^3^Division of Human Genetics, Cincinnati Children’s Hospital Medical Center, Cincinnati, OH, USA

^4^Department of Biomedical Informatics, Cincinnati Children’s Hospital Medical Center, Cincinnati, OH, USA

^5^Departments of Environmental Health and Electrical Engineering & Computer Science, University of Cincinnati, Cincinnati, OH, USA

***Correspondence**:

Vidya Chidambaran, MD

[vidya.chidambaran@cchmc.org](mailto:vidya.chidambaran@cchmc.org)

**Key Words:** Chronic post-surgical pain; systems biology; gene enrichment; genetics; polygenic risk score;

**Supplementary figure legends**

Supplementary Figure 1: Quality control flow diagram and selection of variants on exome chip for analyses. This flow diagram details selection of case and control variant sets to be included for association analyses with chronic post-surgical pain. Variants annotated to chromosome 0, sex chromosomes. Mitochondrial, indels and those without annotation were excluded. Other were excluded based on quality control criteria (minor allele frequency<10%, not satisfying hardy weinberg equilibrium (p>0.0001) and call rate <90%. The final case and control sets, after pruning for linkage disequilibrium were included in enrichment analyses.

Supplementary Figure 2: Recruitment timeline for the spine surgery study cohort is delineated. Of 315 eligible patients who satisfied inclusion/exclusion criteria, reasons for not enrolling and derivation of final cohort (N=131) included in the study with chronic pain outcomes are described.

| **Supplementary table 1**. Literature derived training gene lists for chronic post-surgical pain used to identifying candidate genes and in gene enrichment. |
| --- |
| \| **Gene Symbol** \| **Gene Name** \| \| --- \| --- \| |
| \| *ABCB1* \| ATP binding cassette subfamily B member 1 \| \| --- \| --- \| \| *ATXN1* \| ataxin 1 \| \| *BDNF* \| brain derived neurotrophic factor \| \| *CACNG2* \| calcium voltage-gated channel auxiliary subunit gamma 2 \| \| *CHRNA6* \| cholinergic receptor nicotinic alpha 6 subunit \| \| *COMT* \| catechol-O-methyltransferase \| \| *CTSG* \| cathepsin G \| \| *DRD2* \| dopamine receptor D2 \| \| *GCH1* \| GTP cyclohydrolase 1 \| \| *HLA-DQB1* \| major histocompatibility complex, class II, DQ beta 1 \| \| *HLA-DRB1* \| major histocompatibility complex, class II, DR beta 1 \| \| *HTR1A* \| 5-hydroxytryptamine receptor 1A \| \| *HTR2A* \| 5-hydroxytryptamine receptor 2A \| \| *IFNG* \| interferon gamma \| \| *IL10* \| interleukin 10 \| \| *IL13* \| interleukin 13 \| \| *IL1R1* \| interleukin 1 receptor type 1 \| \| *IL1R2* \| interleukin 1 receptor type 2 \| \| *IL1RN* \| interleukin 1 receptor antagonist \| \| *IL4* \| interleukin 4 \| \| *KCNA1* \| potassium voltage-gated channel subfamily A member 1 \| \| *KCND2* \| potassium voltage-gated channel subfamily D member 2 \| \| *KCNJ3* \| potassium voltage-gated channel subfamily J member 3 \| \| *KCNJ6* \| potassium voltage-gated channel subfamily J member 6 \| \| *KCNK3* \| potassium two pore domain channel subfamily K member 3 \| \| *KCNK9* \| potassium two pore domain channel subfamily K member 9 \| \| *KCNS1* \| potassium voltage-gated channel modifier subfamily S member 1 \| \| *NFKB1* \| nuclear factor kappa B subunit 1 \| \| *OPRM1* \| opioid receptor mu 1 \| \| *P2RX7* \| purinergic receptor P2X 7 \| \| *PRKCA* \| protein kinase C alpha \| |
