# Supplementary figures and images for: Systems Biology Guided Gene Enrichment Approaches Improve Prediction of Chronic Post-surgical Pain After Spine Fusion
Source: Front Genet. 2021 Mar 23;12:594250. doi: 10.3389/fgene.2021.594250 (PMC8044807; doi:10.3389/fgene.2021.594250)

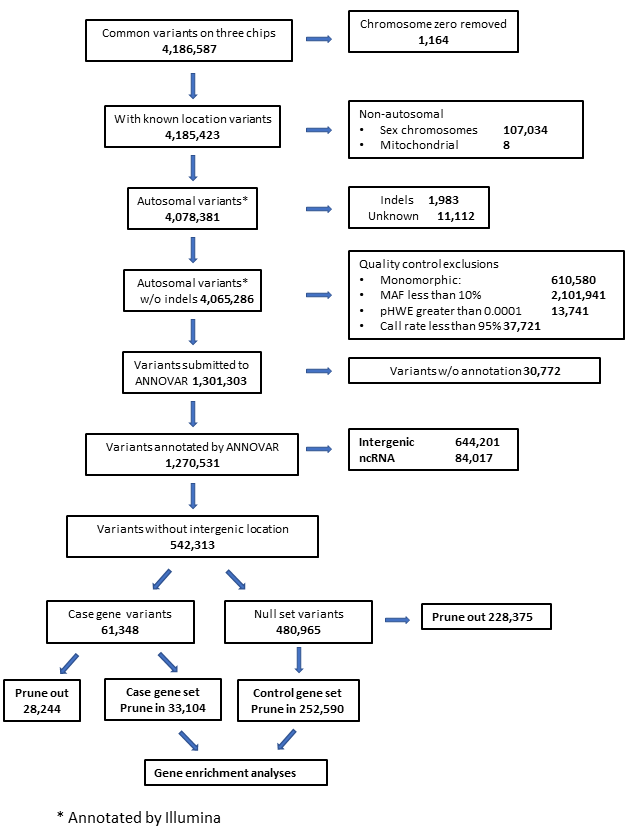

Supplement: Supplementary file 2 [file Image_1.TIF]

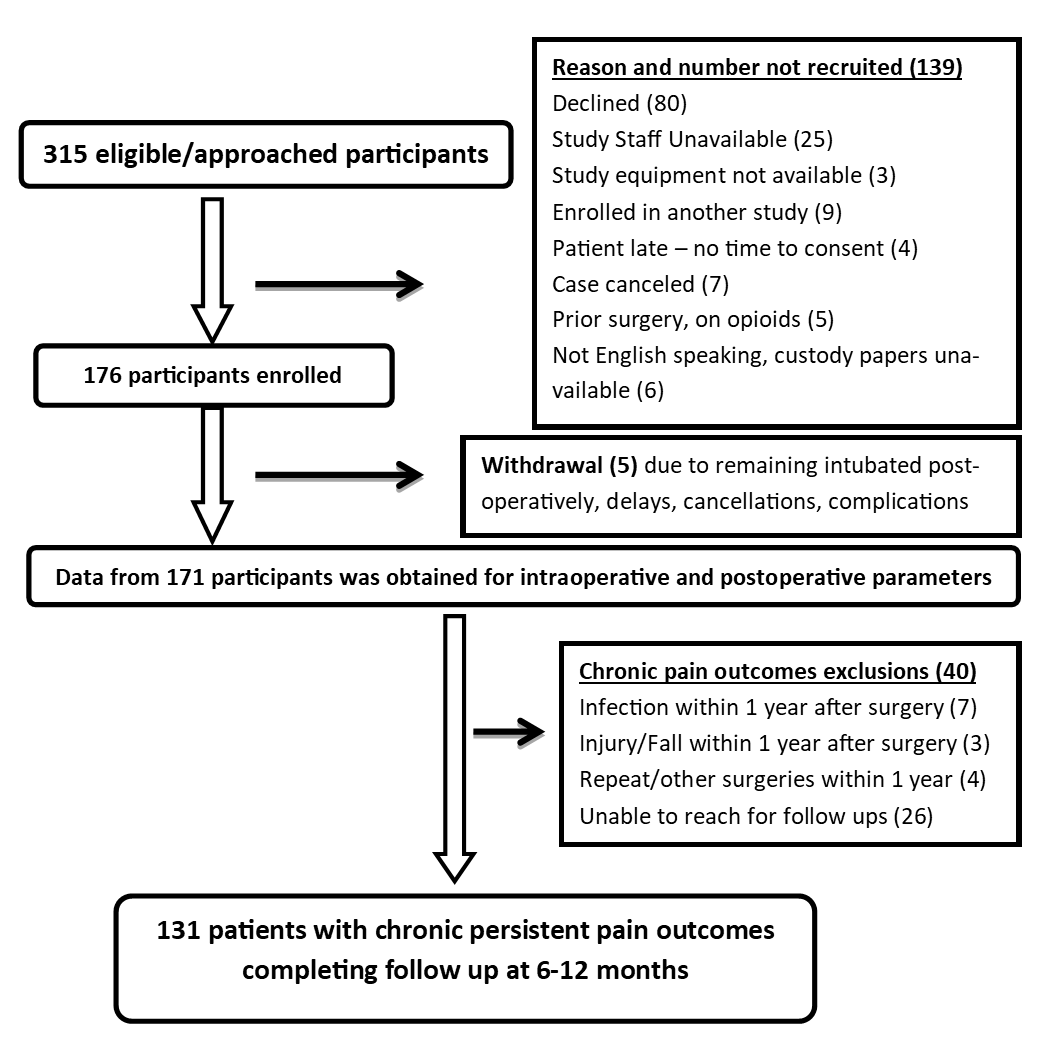

Supplement: Supplementary file 3 [file Image_2.TIF]
